# Emerging Antifungal Resistance in Fungal Pathogens

**DOI:** 10.1007/s40588-024-00219-8

**Published:** 2024-03-18

**Authors:** Sui Ting Hui, Hugh Gifford, Johanna Rhodes

**Affiliations:** 1 MRC Centre for Global Infectious Disease Analysis, Imperial College London, London, UK; 2 MRC Centre for Medical Mycology, University of Exeter, Exeter, UK; 3 Department of Medical Microbiology, Radboudumc, the Netherlands

**Keywords:** Antifungal drug resistance, Public health, *Aspergillus fumigatus*, *Trichophyton indotineae*, *Candida auris*, One Health

## Abstract

**Purpose of Review:**

Over recent decades, the number of outbreaks caused by fungi has increased for humans, plants (including important crop species) and animals. Yet this problem is compounded by emerging antifungal drug resistance in pathogenic species. Resistance develops over time when fungi are exposed to drugs either in the patient or in the environment.

**Recent Findings:**

Novel resistant variants of fungal pathogens that were previously susceptible are evolving (such as *Aspergillus fumigatus*) as well as newly emerging fungal species that are displaying antifungal resistance profiles (e.g. *Candida auris* and *Trichophyton indotineae*).

**Summary:**

This review highlights the important topic of emerging antifungal resistance in fungal pathogens and how it evolved, as well as how this relates to a growing public health burden.

## Introduction

Fungi encapsulate a wide range of diversity, with an estimated 3.8 million species within this kingdom [1] and are currently classified into eight phyla: zoosporic fungi (Cryptomycota, Chytridiomycota and Blastocladiomycota), Microsporidia, Zoopagomycota, Mucoromycota and Dikarya fungi (Ascomycota and Basidiomycota). Reports of emerging pathogens in all phyla are on the increase, due to altered pathogenicity or availability of new biological niches [2]. In addition, there are increased reports of antifungal drug resistance amongst emerged human fungal pathogens and is often found in emerging fungal pathogens. For example, *Cryptococcus gattii* has experienced a shift in species distribution to the Pacific Northwest (PNW) [3], and more recently, *Candida auris* has emerged as an important nosocomial infection [4] on all continents except Antarctica [5]. Fungi demonstrate complex life cycles, enabling genomes to undergo recombination in sexual and parasexual life cycles, and clonal propagation during asexual reproduction. This results in novel genetic diversity and expansion of pathogenic lineages capable of overcoming host defences, survival in non-endemic regions and drug resistance.

The overall burden of fungal disease equates to more than a billion people worldwide [6], causing equally diverse diseases, from superficial infections and allergic syndromes to life-threatening invasive diseases. Treatment of disease is fought with four antifungal drug classes in our arsenal: azoles, echinocandins, polyenes and pyrimidine analogues. Yet clinicians and mycologists alike may say we are losing the battle, with rising drug resistance and tolerance causing treatment failure [7]. The increasing public health threat posed by resistance and treatment failure has been recognised by the World Health Organisation (WHO) [8••] and the US Centers for Disease Control and Prevention (CDC) [9]. A Global Action Plan [10] to address antimicrobial resistance has been endorsed by the WHO and stresses the importance of a One Health approach to tackle it. This plan has called for coordination between human and veterinary medicine, as well as agricultural, environmental and financial sectors, and has certainly helped raise antifungal drug resistance to the top of the global public health agenda.

In this review, we focus on emerging drug-resistant fungal pathogens as well as previously susceptible fungi that have recently evolved resistance and the potential contributors to this evolution.

## Emerging Antifungal Drug Resistance in Previously Susceptible Pathogens

Whilst present in a variety of environments, including soil, compost and decaying organic matter, the filamentous mould *Aspergillus fumigatus* can also cause acute and chronic forms of invasive aspergillosis in immunocompromised individuals [11]. With over 300,000 life-threatening infections in susceptible patient cohorts each year, azole antifungal drugs are the most effective treatment [12]. However, over the last two decades, clinicians have seen a rise in the prevalence of azole-resistant *A. fumigatus* in patients, mirroring a rise in azole resistance environmentally [13]. Whilst initially responsive to azole antifungal drug therapy, susceptibility has given way to resistance.

It is thought that at least two-thirds of patients with azole-resistant infections have not received azole drug therapy [14], suggesting an environmental route of acquisition [15, 16]. The most prominent set of polymorphisms conferring resistance to azoles is the 34 base pair (bp) tandem repeat and L98H amino acid substitution in *cyp51a*, TR_34_/L98H (Table 1), which has been recovered both within clinical samples and environmentally. Much research has been conducted to establish whether azole-resistant *A. fumigatus* has an agricultural origin [17, 18] or evolved independently in the clinic [19].

**Table 1:** Known drug resistance mechanisms for emerged and emerging fungal pathogens *A. fumigatus*, *C. auris* and *T. indotineae* for the four main classes of clinically used antifungal drugs

Species	Azoles	Echinocandins	Polyenes	5-flucytosine	Allylamines
*Aspergillus fumigatus*	*Cyp51A* amino acid substitutions (L98H, M220, G54, Y121F/T289A) *Cyp51A* promoter tandem repeats (TR_34_, TR_46_, TR_53_, TR_120_)	*FKS1* amino acid substitution (S678P)	*ERG3* amino acid substitutions	pH-dependent downregulation of *FCY1*	
*Candida auris*	*ERG11* amino acid substitutions (Y132F, K143R, F126L)	*FKS1* amino acid substitutions (S639F/P/Y, F635C)	*ERG6* indel mutation	*FUR1* amino acid substitutions (F211I) *FUR1* partial deletion *FCY1*/*FCY2* amino acid substitutions (S70R, M128fs)	
*Trichophyton indotineae*	*ERG11* promoter tandem repeats (hypothesised) *Cyp51B* overexpression *ERG1* amino acid substitution (A448T)				*ERG1* amino acid substitutions (F397L, L393F, F397L/A448T)

If azole-resistant *A. fumigatus* isolates originated in the environment and were subsequently acquired by patients, it would be logical to assume clinical isolates would show resistance to agricultural fungicides. Kang et al. showed clinical *A. fumigatus* isolates carry alleles conferring resistance to agricultural fungicides such as benzimidazoles (MBCs) and quinone outside inhibitors (QoIs) [20••]; this subsequently provides strong evidence for an agricultural origin of isolates acquired by patients. This finding was quickly followed by a study by Rhodes et al. that showed multiple clinical isolates with azole drug-resistant genotypes that matched environmental isolates with very high similarity [21••]. Since no evidence has been presented to convincingly show patients transmit *A. fumigatus* into the environment, it is further evidence for the acquisition of azole-resistant isolates by at-risk patients from the environment. Other fungal diseases have been shown to be acquired from the environment, such as *Coccidioides* spp. [22] and *Cryptococcus* spp. [23], further bolstering support for this argument.

*A. fumigatus* not only represents a human public health threat, but also a threat to wildlife and conservation efforts. Recently, a single strain of *A. fumigatus* was the cause of a mass aspergillosis outbreak in an endangered parrot species [24]. Despite not being drug-resistant, the outbreak still resulted in 21 infections and 9 deaths, in a population of only 211. A known opportunistic pathogen of penguins [25], azole drug-resistant *A. fumigatus* has been isolated from a zoo environment and from Humboldt penguins themselves [26]. However, little research is dedicated to the impact of aspergillosis on wildlife species in terms of clinical outcome and treatment failure, certainly highlighting a gap in our current knowledge.

Given the above evidence, it is likely that resistance has emerged in the previously susceptible *A. fumigatus* due to the dual use of azoles in the environment and the clinic, driving the evolution of antifungal resistance [2]. The TR_34_/L98H resistance allele, along with TR_46_/Y121F/T289A (Table 1), is associated with itraconazole and voriconazole resistance respectively and has been isolated within and outside the clinic [27]. A contributing factor for the generation of novel drug resistance alleles could be attributed to the high recombination rate observed in *A. fumigatus* [28]; a recent example of a pan-azole resistance phenotype, attributed to TR_34_/L98H/T289A/I364V/G448S, could be the result of such recombination [29]. Thus, the pandemic potential of drug-resistant *A. fumigatus* has already been demonstrated, primarily due to human activity by using clinical antifungals in horticultural products [30], and global spread is likely due to ease of dispersal via conidia on air currents [2].

## Emerging Drug-Resistant Fungal Pathogens

We have recently witnessed entirely new fungal species emerge and spread globally, already resistant to multiple antifungal drugs. *Candida auris* has received much public attention and scientific research focus and was recently listed as a “critical priority” fungal pathogen in the inaugural WHO Fungal Priority Pathogen List (FPPL) [8••]. Fungicide exposure and climate change have been hypothesised as factors explaining the rapid worldwide emergence of *C. auris*, which displays high intrinsic levels of fluconazole resistance and varying levels of resistance to other antifungal drugs [31].

First described in 2009 in Japan [4], *C. auris* has since been reported in over 40 countries [32]. Initial genomic analyses revealed four geographically restricted clades emerging almost simultaneously, I (South Asia), II (East Asia), III (South Africa) and IV (South America) [33], which have since been found elsewhere in the world. Interestingly, isolates from Clade II do not tend to cause invasive infections and are susceptible to antifungal agents. In recent years, a further two new clades, V from Iran [34, 35] and VI from Singapore [36], have also been described. Yet unlike *A. fumgiatus*, *C. auris* has emerged with intrinsic fluconazole antifungal drug resistance [37] and has since evolved drug resistance to other azoles and drug classes [38••].

*C. auris* possesses different characteristics to set it apart from other *Candida* species, such as a lack of morphological switching between yeast and hyphal forms [39, 40], which may explain colonisation of distinct niches. For example, *C. auris* primarily colonises the skin, whereas *C. albicans* colonises and infects mucosal surfaces [37]. Antifungal drug tolerance is attributed to biofilm formation in *Candida* species due to drug sequestration by the biofilm mannan-glucan polysaccharide matrix [41] and plays a key role in intrinsic resistance in *C. auris* [33, 42, 43]. Previous studies have shown that Clade II isolates seem to have evolved host adaptation and are particularly adapted to the ear, resulting in non-invasive clinical phenotypes that are drug-susceptible [44] and incapable of forming biofilms [45].

Whole genome sequencing has helped unravel the basis of the resistance mechanisms existing and evolving within *C. auris* (Table 1). The main cause of fluconazole resistance and therefore treatment failure is centred on amino acid substitutions within the *ERG11* gene, which are also restricted by genetic clade: Y132F and K143R in Clade I, only Y132F in Clades IV and V and F126L in Clade III [33, 35]. Currently, no known mutations in *ERG11* have been associated with Clade VI isolates, but given the susceptibility of all three identified isolates in this clade, it remains unlikely any will be found [36]. Similarly, given the drug susceptibility of Clade II isolates, no *ERG11* mutations are associated with this clade [33]. Mutations within *ERG11* account for the majority of azole drug resistance and were initially attributed as the causation of intrinsic fluconazole resistance. Mutations caused by amino acid substitutions have also been described in *TAC1b* [46], along with overexpression of *CDR1* and *MDR1* [47], but the necessary experiments to confirm these contributions have not yet been carried out in all cases.

Since the first genomic analyses were completed on *C. auris* in 2017 by Lockhart et al. [33], more than 5000 genomes have been made publicly available. Genomic data has allowed the mining of confirmed and probable mutations linked to antifungal drug resistance (Table 1). Of greatest abundance are the clade-specific *ERG11* mutations conferring fluconazole resistance. Yet over time, mutations conferring resistance to other antifungal drug classes have been described: the first European outbreak detailed flucytosine resistance due to F211I in *FUR1* and echinocandin resistance due to S639Y in *FKS1* [48]. This later mutation is situated in the hotspot 1 (HS1) region of *FKS1*, and other amino acid substitutions (S639P and S639F) have been described at the same loci [49, 50]. Recently, pan-drug class resistance has been described in a single patient undergoing multi-visceral transplantation, with *ERG11* K143R, *FKS1* S639Y and a novel *FUR1*[1Δ33] mutation [38••]. This combination conferred resistance to azoles, echinocandins and flucytosine, respectively. In addition, resistance to amphotericin B was also observed, yet no distinct variants were identified, suggesting unknown genetic elements were driving resistance to this drug. The recent emergence of a *C. auris* isolate resistant to four classes of antifungal drugs is concerning; further research is needed to ascertain patient outcome consequences and the mechanism(s) behind novel drug resistance mutation evolution.

The question of how resistance emergences in *C. auris* has been explored through a combination of *in vitro* microevolution and *in silico* genomic approaches. Serial drug exposures have shown rapid adaptation leading to tolerance of echinocandins (*FKS1* codon deletion and mutations in *FKS1, ERG3* and *CIS3*), azoles (chromosome 5 aneuploidy, containing *TAC1b*, *ERG11* contig duplication, mutations in *TAC1b*), polyenes (*MEC3* mutations) and concurrently azoles and polyenes (*ERG3/ERG11* nonsense mutations) [51]. Sub-telomeric deletions, aneuploidies and SNP acquisitions were also seen on fluconazole exposure with evidence of a potential hypermutator phenotype resulting in missense or nonsense impact mutations in one strain with an associated *MLH1* variant (A216V) [52]. However, evidence for recombination in *C. auris* has not been convincingly demonstrated since major clades diverged, according to testing for phylogenetic incompatibility and linkage equilibrium testing [53]. The use of bioinformatic approaches in public repository data is limited by the availability of phenotypic data, which might be used in genome-wide association studies. One group used 356 strains to test several machine learning clustering methods to rank the contribution of specific mutations to resistance, including in several genes that have not been explored or described [54].

More recently, *Trichophyton indotineae* has emerged as a novel dermatophyte, posing a significant health threat in India due to its extensive infection and treatment failures [55, 56]. Fungal infections caused by dermatophytosis that affect the skin and nails and have a global prevalence of 20–25% [57]. Initially described as *Trichophyton mentagrophytes* genotype VIII, *T. indotineae* has recently been classified as a distinct species with unique characteristics, including high terbinafine (TERB) resistance and anthropophilic transmission [55].

*T. indotineae* causes highly inflammatory, often widespread, dermatophytosis affecting much of the lower body, groins, limbs and face [55]. The emergence and isolation of this species took place sequentially, starting in Australia in 2008, then spreading to Oman in 2010, and later to Iran in 2016 [55]. Around 2017–2018, a drug-resistant variant of *T. indotineae* emerged in India, setting off a global spread from the Indian subcontinent [55, 56]. Reported cases are seen in several countries, including India, Bahrain, United Arab Emirates, Oman, Iran, Germany, France, Italy, Belgium, Switzerland, Greece, Denmark, Finland, China, Australia, Canada, Japan, Vietnam and the United States of America [55, 58–69].

The development of resistance in *T. indotineae* is primarily linked to the excessive and improper use of over-the-counter corticosteroid-antifungal medications [70]. As such, *T. indotineae* now exhibits a high frequency of resistance to conventional antifungals, including allylamines (which includes TERB) and azoles (itraconazole and fluconazole) [71–74].

TERB interacts with squalene epoxidase (SQLE), a key enzyme encoded by the *ERG1* gene, in which SQLE plays a pivotal role in initiating the biosynthesis of ergosterol [75, 76]. Mutations in the *ERG1*/*SQLE* gene at specific amino acid positions, such as Phe397Leu (F397L) (associated with minimum inhibitory concentration (MIC) values ranging ≥10 to ≥15 µg/mL) and Leu393Phe (L393F) (associated with MIC values in access of ≥40 µg/mL), have been identified to result in high MIC values to TERB [71]. Furthermore, when double mutations occur at Phe397Leu and Ala448Thr (F397L/A448T) within the *ERG1* gene, they result in a combined resistance to TERB [71]. This leads to MIC values that are higher than those observed in wild-type strains or single mutants with the F397L single mutation (with MIC ranges typically falling between 15 to ≥30 µg/mL) [71].

Azoles exert their antifungal effects by binding to the lanosterol 14-α demethylase enzyme, encoded by the *ERG11* gene [71]. These proteins are categorised under the heme-containing monooxygenases within the cytochrome P450 superfamily, *CYP*51 [71]. Thus, point mutations within the *ERG11* gene lead to alterations in amino acid sequences at specific positions, ultimately conferring resistance to particular azole drugs [71, 77, 78]. However, no research has yet detected mutations within *ERG11*. However, the presence of identified tandem repeats in the promoter region and the overexpression of *Cyp51B* suggests the reduced sensitivity of *T. indotineae* strains to azoles [79]. Intriguingly, *ERG1*/*SQLE* mutants carrying the A448T mutation remain resistance-sensitive to TERB but, instead, show azole resistance patterns [71, 73, 80]. The underlying reason for this observed correlation remains undisclosed.

To better understand the TERB mechanisms of resistance in this newly emerging species *T. indotineae*, one study proposes that the diversity of resistance mechanisms may be linked to the *TruMDR2* and *salA* genes, initially identified in other fungal species, thus suggesting promising directions for future research [81]. Our current understanding of *T. indotineae*’s resistance mechanisms is limited, but preliminary findings suggest that *TruMDR2* encodes an ABC transporter known to be part of the multidrug-resistance class of transporters and has been shown to be responsive to various drugs in *T. rubrum* [82]. On the other hand, the *salA* gene, identified in UV-induced TERB-resistant mutants of *Aspergillus nidulans*, encodes a salicylate 1-monooxygenase and contributes to TERB resistance by conferring resistance upon transformation [83]. Hence, the study suggests a similar role in modulating TERB susceptibility in *Trichophyton rubrum* due to its potential involvement in naphthalene ring degradation mechanisms [83]. Further exploration of these genes in *T. indotineae* could provide valuable insights into its unique resistance mechanisms.

## Contributions to the Development of Drug Resistance

Certain fungal species, such as *Candida* spp., exhibit intrinsic drug resistance, yet acquired resistance is also increasing in prevalence. Selection for drug resistance in *T. indotineae* has been attributed to the ease of availability of over-the-counter medications and incomplete treatment courses. The use of low-dosage exposure along with incomplete coverage of topical creams has also been suggested as a contributing factor [84]. Studies have also confirmed the acquisition of fungi from the environment, such as *A. fumigatus* [20••, 21••], *Cryptococcus* spp. [23] and *Coccidioides* [22]; it can therefore be assumed that environmental exposure to antifungal drugs could also contribute to emerging resistance, mirroring the public health threat posed by antibiotic resistance.

Evolutionary pressure demonstrated by the usage of fungicides in the environment will also likely pre-select resistant fungi before they emerge as pathogens of humans. Some azole fungicides used to protect crops from fungal disease have displayed long half-lives in the soil, potentially due to hydrophobic properties: for example, triadimenol has a half-life in the soil varying from 110 to 375 days [85]. Given that chemicals, including azole fungicides, present in topsoil could be washed into waterways [86] and wastewater [87], fungi have the potential to be exposed in many habitats. Fungicide usage is not limited to crop protection, and these chemicals are also used in the horticultural and timber industries [88], providing ample opportunities for fungal communities to be exposed and develop resistance.

Anthropogenic activity will likely contribute to the initial emergence and subsequent dispersal of these pre-selected drug-resistant fungi. Whilst the acquisition of drug resistance is usually associated with reduced capacity for pathogenicity and reduced overall fitness, we are indeed now witnessing highly fit drug-resistant fungal pathogens, e.g. *C. auris*. It is hypothesised that climate change causing a warming planet will also lead to the selection of fungal species capable of infection in humans [31], and anthropogenic activity will change species distribution ranges.

## Conclusion

Since its first description nearly 15 years ago, *C. auris* has now been reported around the world, with the exception to Antarctica, creating a global public health due to high levels of antifungal drug resistance and mortality. At this time, over 1000 publications have been written on *C. auris*, of which nearly 15% feature or mention genomics (PubMed accessed 10^th^ October 2023). The medical and research communities have worked together to not only discover and describe this important pathogen but have also applied cutting-edge technologies to address the question of why *C. auris* has emerged and how it is evolved to further drug resistance. This presents a roadmap for One Health stakeholders to follow for collaboration and intervention to prevent *T. indotineae* from following a similar trajectory to *C. auris*.

Other fungal pathogens will likely emerge, either following the initially susceptible or intrinsically resistant trajectories of *A. fumigatus* and *C. auris* respectively. With continued use of antifungal drugs in the environment and clinically, regardless of the origins of emergence, resistance will be sure to follow. Recent reports have mentioned *Candida africana* as an important emerging agent of vulvovaginal candidiasis [89]; whilst drug resistance is still displaying low prevalence [89], isolated cases are reporting oral candidiasis and altered drug susceptibility profiles [90], perhaps alluding to the potential for a more severe public health problem.

Yet humans are not solely the recipient of drug-resistant fungal pathogens; wildlife and plants are also targets, whether opportunistic or otherwise, thus increasing the opportunity of not only the evolution of further drug resistance, but also “spillover” events into other species. Whilst transmission of a fungal pathogen from the environment to an animal and then on to humans is not a regularly occurring event, there is both potential and precedent for this to occur [91].

As guardians of this planet, humans have a collective responsibility to work together to prevent catastrophe caused by antimicrobial resistance, including antifungal drug resistance. An integrated One Health approach is crucial not only to aid our understanding of the roads leading to drug resistance, but also how we can mitigate the spread across host species at risk of infection. Improved antimicrobial stewardship in medical, agricultural and veterinarian treatment planning is an obvious choice, limiting the use of drug classes for clinical or agricultural usage (and preventing dual-use) and thus slowing the selection pressure and drug resistance evolution. But we can be more ambitious, learning from the success of open-access genomic surveillance platforms generated as part of the COVID-19 pandemic [92], to track the emergence and spread of fungal pathogens globally and across habitats. We can also embrace new technologies, such as rapid “real-time” sequencing for resistance diagnostic test development.

Given the increase in novel emerging fungal pathogens as well as antifungal drug resistance in recent years, a co-ordinated global response between multiple industries is desperately needed. We call on funding agencies to support the creation of these networks to tackle this urgent problem.

## Data Availability

No data is associated with this paper as it is a review.
